# Dengue transmission model by means of viremic adult immuno-competent mouse

**DOI:** 10.1186/1756-3305-7-143

**Published:** 2014-03-31

**Authors:** Lucky Ronald Runtuwene, Eiji Konishi, Atsushi Yamanaka, Yoshihiro Makino, Yutaka Suzuki, Tomohiko Takasaki, Ichiro Kurane, Takashi Kobayashi, Yuki Eshita

**Affiliations:** 1 Department of Infectious Disease Control, Faculty of Medicine, Oita University, Oita 879-5593, Japan; 2 BIKEN Endowed Department of Dengue Vaccine Development, Faculty of Tropical Medicine, Mahidol University, Bangkok 10400, Thailand; 3 Department of Public Health and Epidemiology, Faculty of Medicine, Oita University, Oita 879-5593, Japan; 4 Department of Medical Genome Sciences, The University of Tokyo, Chiba 277-8562, Japan; 5 Department of Virology I, National Institute of Infectious Diseases, Tokyo 162-8640, Japan

**Keywords:** *Aedes aegypti*, Dengue virus type 2, Mass-infection, Viremia, Immuno-competent mouse

## Abstract

**Background:**

Dengue virus infection manifests in three distinct forms in humans: dengue fever, dengue hemorrhagic fever, and dengue shock syndrome. Infection with the virus is a fatal disease; no vaccine is available and prevention depends on interruption of the chain of transmission. The study of dengue viral transmission by mosquitoes is hindered due to the lack of an affordable animal model. In general, immuno-competent mice are used as a simple and inexpensive animal model, but mice are not susceptible to dengue virus infection and therefore viremia will not occur following the inoculation of the virus in such mice. Here, we report a method for creating artificial viremia in immuno-competent mice, and further demonstrate the use of viremic mice to simultaneously infect a large number of *Aedes aegypti.*

**Methods:**

We infected K562 cells with DENV-2 in the presence of an antibody against DENV-4. We then incubated the cells for 2 d before injecting the infected cells into C3H mice. After 5 h incubation, we allowed 100–150 female *Aedes aegypti* to feed on blood from the mice directly. We collected blood samples from the mice and from randomly selected *Ae. aegypti* at 2, 6, 12, and 24 h post-blood meal and screened the samples for DENV-2 genome as well as for virus concentration.

**Results:**

Our procedure provided high virus concentrations in the mice for at least 7 h after viral inoculation. We found that 13 out of 14 randomly picked mosquitoes were infected with DENV-2. High concentrations of virus were detected in the mosquitoes until at least 12 h post-infection.

**Conclusions:**

Using the viremic immuno-competent mouse, we show that mass infection of *Ae. aegypti* is achievable. Compared to other infection techniques using direct inoculation, membrane-feeding, or immuno-deficient/humanized mice, we are confident that this method will provide a simpler and more efficient infection technique.

## Background

Dengue virus (DENV) is among the deadliest arthropod-borne disease in the world. The virus causes dengue fever and its more severe forms, dengue hemorrhagic fever and dengue shock syndrome. The disease has a global distribution, with South East Asia and Western Pacific regions carrying 75% of the current global disease burden [1]. In endemic areas, cases are reported year-round, with a surge of incidence observed during intervals of increased rainfall. During wet periods, the principal disease vector, *Aedes aegypti*, and a secondary vector, *Ae. albopictus*, increase in range of habitat. The close proximity of the DENV vector to human populations permits easy transmission of the virus from one infected person to another [2]. Disease control is complicated by the fact that no vaccines or specific medicines are available for prevention or treatment of the disease. Hence, DENV poses an imminent danger that can seriously compromise public health in heavily populated tropical urban areas. It is complicated by the poorly understood factors of hyperendemic, community, and inter-annual transmission [3]. However, characterization of these factors and their relative importance on transmission has been complicated by the lack of a suitable laboratory model for DENV transmission [4].

Understanding dengue transmission has always been difficult due to the lack of animal models. Viremia is a feature required in a dengue-vector transmission experiments. The minimum virus concentration needed to cause infection in mosquitoes has not been formally defined. Previous reports indicated that doses of 4 × 10^4^ PFU/ml (in chick-skin membrane) [5] and 5 × 10^4^ PFU/ml (intravenous injection in adult rhesus monkey) [6] sufficed for transmission, suggesting that concentrations of approximately 10^4^ PFU/ml represent a lower limit for effective infection in mosquitoes. This minimum dose is achievable by only three naturally occurring hosts that can naturally amplify DENV: mosquitoes, humans, and non-human primates (NHP) [7]. NHP, including the common marmoset, can develop viremia that manifests as very mild or no clinical signs of disease [8,9]. However, research using NHP remains a challenge for many researchers, given the high cost and limited accessibility of such models [10]. Furthermore, the utilization of NHP for transmission experiments might be considered a waste of resources. Non-costly and easily accessible animals are preferable for such experiments.

Mice are generally considered the animal of choice for many experiments due to the fact that they are small and inexpensive to maintain. However, wild type mice, i.e. immuno-competent mice, such as A/J, BALB/c, C57BL/6 are only transiently permissible to DENV replication [11-13]. A/J mouse showed a slight viremia on day 2 post DENV-2 injection, shown only by RT-PCR [12]. BALB/c showed an undetectable level of DENV-2 viremia, which stands below the sensitivity assays for normal virus titre and/or isolation [13]. Immuno-competent mice exhibited peripheral viremia only when suckling wild-type mice were injected with DENV intracranially [14]. Peripheral replication of DENV in mice was first reported in either immuno-deficient or humanized mice [15-17]. Mice that have had their immunity disabled, such as Interferon Regulatory Factor (IRF) 3/7 knock-out mice, have been shown to support DENV-2 replication and to be transmitted to mosquitoes [15]. Interferon (IFN) receptor-deficient mice (AG129) lack the receptors for IFNα/β and γ, permitting all four DENV serotypes to replicate after peripheral inoculation [16]. Subcutaneous infection of humanized NOD/SCID mice with low-passage dengue human isolates also results in measurable viremia [17].

To date, dengue-vector transmission experiments have been performed by direct intrathoracic inoculation of the virus, artificial feeding technique, or using viremic animal models, e.g., humans, suckling mice, or humanized mice [14-20]. The disadvantages of utilizing direct inoculation and membrane-feeding techniques are that both procedures are time-consuming and laborious, especially for the production of large numbers of infected mosquitoes. The techniques are also unnatural, as they do not involve live animals. Newborn wild-type mice have been used for transmission of dengue to vector, but they required intracranial injection of high dose DENV at less than 24-hour-old [14]. Viremic humanized mice have also been used for transmission of dengue to vector [20], but maintenance of these animals requires extra resources. Consequently, immuno-competent mice are the preferred vessel for large transmission experiments, especially if resources are limited.

In this paper, we develop a method to produce viremia in immuno-competent mice. We were able to maintain high virus concentration in the mice until at least 7 h post-injection. Within this window period, we managed to infect a large number of adult female mosquitoes simultaneously from a single mouse host.

## Methods

### Materials

*Ae. aegypti* Liverpool Inbreeding (INB12) strain was provided by Dr. Akio Mori, University of Notre Dame, Indiana, USA. Five-week-old C3H mice were purchased from Japan SLC, Japan. K562 erythroleukemia cells were purchased from Dainippon Sumitomo Pharma, Japan. Vero cell cultures were acquired from the American Type Culture Collection (ATCC). DENV-2 ThNH7/93 strain was kindly provided by Dr. Akira Igarashi from the Institute of Tropical Medicine, Nagasaki University. Monoclonal antibody (mAb) against DENV-4 (D4-I-1D6) was obtained from Dr. Eiji Konishi, Kobe University.

### Cell infection with DENV-2 in the presence of mAb against DENV-4

**Figure 1:**
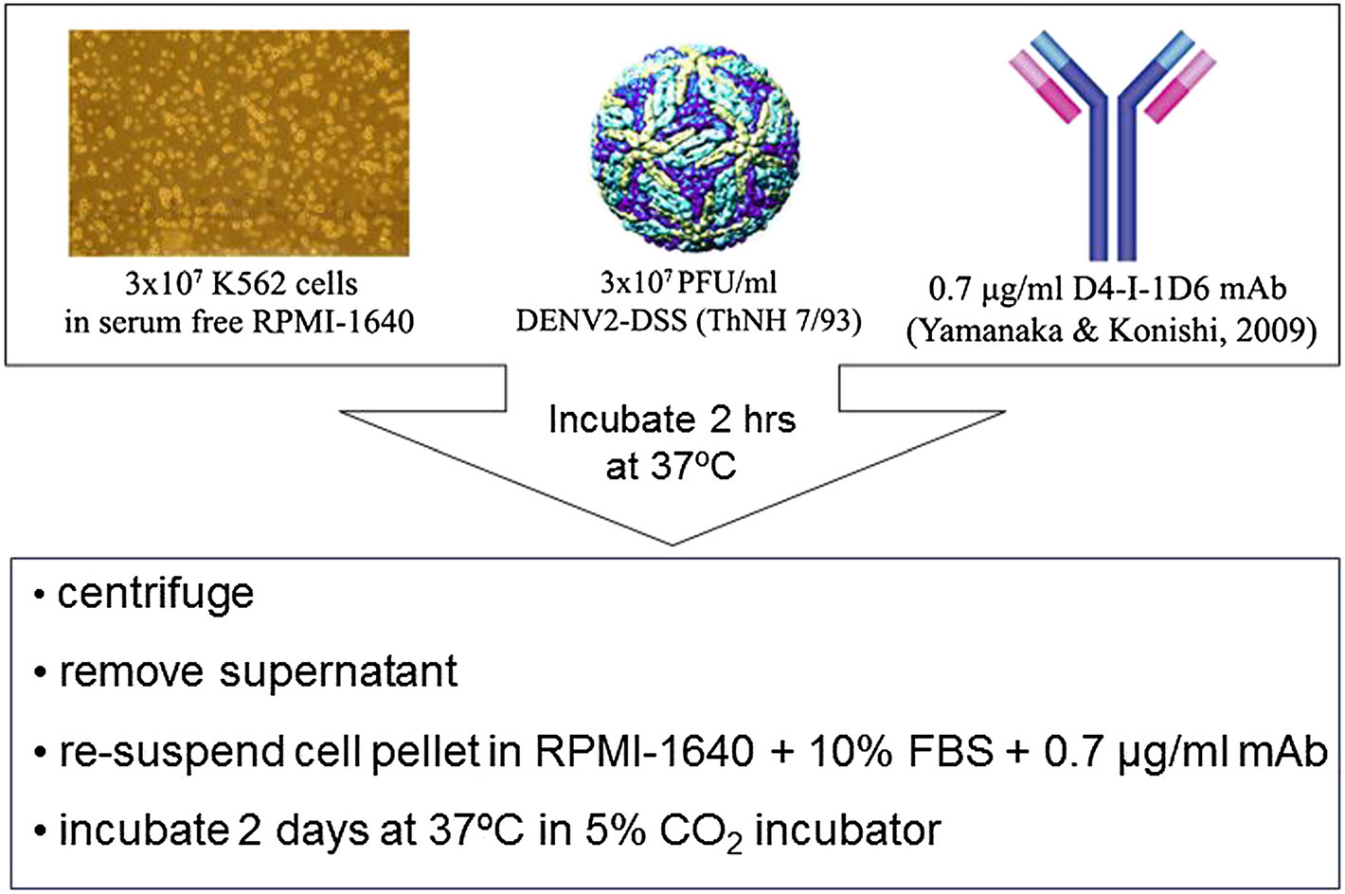
**DENV-2 infection of K562 cells.** Prior to infection of mice and mosquitoes, DENV-2 is propagated in K562 cells at MOI 1.0 in the presence of antibody against DENV-4. After incubation for 2 h in 37°C, cells are centrifuged, then resuspended in an equivalent volume of fresh medium containing antibody against DENV-4. The mixture then is incubated for two days at 37°C.

K562 erythroleukemia cells were grown in RPMI-1640 medium (Sigma-Aldrich) supplemented with 10% HyClone (Thermo Scientific). Cells were cultured in a humidified atmosphere containing 5% CO_2_ at 37°C until a density of 1.5 × 10^7^ cells/mL per aliquot was reached. The cells then were infected with 1.5 × 10^7^ PFU/ml DENV-2 ThNH7/93 (MOI 1.0). The suspension was supplemented to 0.7 μg/ml with D4-I-1D6 mAb. After incubation for 2 h in 5% CO_2_ at 37°C, the infected cells were collected by brief centrifugation and re-suspended in RPMI-1640 supplemented with 10% HyClone and 0.7 μg/ml of D4-I-1D6 mAb. The final suspension then was transferred into filter-cap flasks and incubated in 5% CO_2_ at 37°C for 2 d (Figure 1).

### Mouse infection and blood collection

After 2 d of incubation, the suspension was centrifuged briefly. The supernatant was removed and the pellet was re-suspended in RPMI-1640 medium supplemented with 10% FBS and centrifuged a second time. The supernatant was again removed and the pellet was resuspended in 1 ml of serum-free RPMI-1640 medium and sub-divided into four equal volumes. Using a 1-ml disposable syringe fitted with an 18-G needle, each aliquot was aspirated into syringe. Each aliquot of cell suspension was injected intraperitoneally (via 22-G needle) into 5-week-old C3H mice. Blood samples were collected at 4, 5, 6, and 7 hours post-infection from the retro-orbital sinus according to standard method [21] and then subjected to plaque assay (below).

### Mosquito infection and harvesting

*Ae. aegypti* Liverpool (INB12) strain was reared in a controlled environment: temperature was 26 ± 1°C, relative humidity was 80–95%, and LD cycle of 16:8 was maintained daily. Larvae were fed a combination of a finely-ground mouse food (CLEA, Japan) and fish food (Tetramin, Germany). Pupae were collected into plastic cups until emergence. Emerged mosquitoes were transferred into a mesh-cage with unlimited access to 4% sugar solution until 5–9 d old. Mosquitoes were not blood-fed prior to experiment. The mosquitoes were given a chance to feed from the infected mice at 5 h post-mouse infection. The mice were anesthetized and placed on top of the cage. The mosquitoes were allowed to feed for 1 h; about 100–150 female mosquitoes fed from a mouse. After 1 h, the mosquitoes were incubated in a 28°C incubator. Mosquitoes were sacrificed 2, 6, 12, or 24 h post-feeding by flash-freezing. The carcasses were kept at -80°C prior to processing. The study was carried out in strict accordance with the recommendations of The Committee for Animal Experimentation, Oita University, at the P3 laboratory of the Oita University Animal Center.

### Confirmation of infection

Confirmation of infection was performed using two methods: plaque assay and reverse transcriptase polymerase chain reaction (RT-PCR). In the plaque assay, four mosquitoes were randomly picked from each time point. Mosquitoes were individually ground in 100 μl MEM (Nissui) supplemented with 2% HyClone. The solution was reserved as the undiluted (10°) inoculum. The supernatant was diluted 10^1^- to 10^6^-fold and 100 μl of the diluted supernatant was transferred into separate wells of 12-well-plate (Corning) layered with confluent Vero cell monolayers. After 1 h of incubation at 37°C in a 5% CO_2_ incubator, the infected monolayer was layered with 2% methylcellulose and incubated again in 37°C, 5% CO_2_ incubator for 9 d. The gel-like solution was removed after 9 d. The monolayer was fixed with formalin and stained with 1.25% trypan blue. The plaques were counted and the results were normalized as PFU per mosquito. To check infectivity with RT-PCR, supernatant from ground mosquitoes was first purified using RNeasy Mini Kit (Qiagen). The purified RNA then was subjected to RT-PCR using SuperScript III One-Step RT-PCR System with Platinum Taq (Invitrogen) according to company’s protocol [22] using previously published universal DENV primers [23].

### Statistical analysis

Difference in virus titers in the mice among each time point were analyzed using repeated measures ANOVA after log transformation. Virus titers in mosquitoes were compared among each time point using one-way ANOVA after log transformation [log (x + 1)]. Statistical analyses were performed using the JMP 11.0.0 software package (SAS Institute).

## Results

### Mass infection of *Ae. aegypti* is achievable

**Figure 2:**
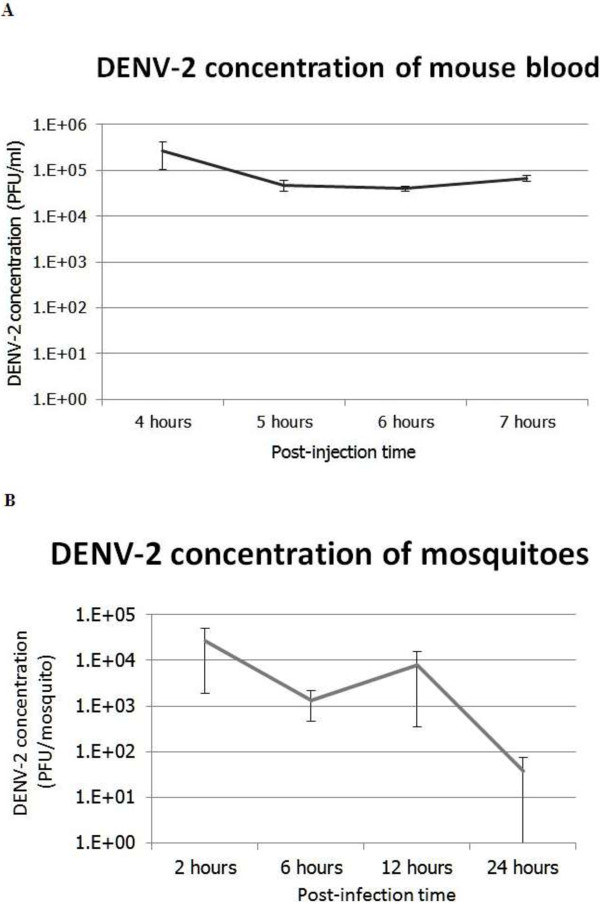
**The measurement of DENV-2 concentration in infected C3H mouse blood (A) dan early-stage infected mosquitoes (B).** DENV-2 titer from infected C3H mice **(A)**. DENV-2 titer after virus inoculation to mice intra-peritoneally using our technique. The blood was obtained from the orbital sinus and subjected to plaque assay. The result is the mean (±SE) of three different plaque assay experiments for each time point. DENV-2 titer from early-stage-infected mosquitoes **(B)**. DENV-2 titer after oral virus administration on mosquitoes using our technique. Mosquitoes were randomly picked from a colony of 100–150 females and subjected to plaque assay. The result is the mean (±SE) of three to four individual mosquitoes for each time point.

In order to achieve high levels of viremia sufficient to cause infection of mosquitoes, an antibody was first used to enhance virus replication within cells. This method exploits the characteristic of dengue virus to enhance replication in the presence of an antibody against different serotypes. These infected cells were then injected into an immuno-competent mouse, permitting the maintenance of a high virus titer for at least 7 h post-injection and no significant difference was observed among each time point (Figure 2A; repeated measures ANOVA: d.f. = 3, *F* = 3.2, *P* = 0.1.036). Within this time period, the mosquitoes were allowed to feed from the viremic mouse. The virus was viable and achieved a high titer in the mosquitoes’ bodies except on 24 h post-feeding (Figure 2B; one-way ANOVA: d.f. = 3, *F =* 8.6, *P* = 0.0049).

### Most of the mosquitoes are infected

**Figure 3:**
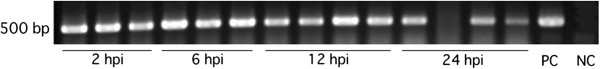
**RT-PCR of infected mosquitoes.** DENV-2 confirmation of infected mosquitoes at various time points shows that 13 out of 14 mosquitoes were infected, as shown by the existence of 500 bp band using RT-PCR. 2 hpi: 2 h post-infection, 6 hpi: 6 h post-infection, 12 hpi: 12 h post-infection, 24 hpi: 24 h post-infection, PC: positive control, NC: negative control.

RT-PCR was used to confirm the infection. The RNAs of three to four mosquitoes that were randomly picked from each time point were purified using RNeasy Mini Kit according to the company’s protocol. Using DENV-2 universal primers, RT-PCR gave 13 positive results out of 14 tested samples, or 93% positive (Figure 3).

## Discussion

Viremia is a feature required for dengue-vector transmission experiment. NHP and immuno-deficient mice have been shown to easily display viremia, but the price, limited accessibility, and reduced practicality of these animal models make them less attractive. Consequently, immuno-competent mice are the preferred vessels for transmission experiments. Except for newborn mice, however, these mice do not easily develop viremia post-dengue infection; hence it is difficult to use these mice in simple transmission experiments. Therefore, we have developed a method to orally infect a large number of mosquitoes by means of viremic adult immuno-competent mouse using a combination of cultured cells, DENV, and monoclonal antibody.

Dengue virus is unique in that the antibody developed due to exposure of the virus *in vivo* provides only a limited interval of protection against other serotypes of virus. Once the threshold is lower than a certain level, the antibody binds to the different serotype of virus with sub-neutralizing properties instead of neutralizing, i.e. the virus is still viable for replication [24]. The virus still gains access to monocytic cells via the Fc receptor and propagates, causing enhancement of viremia. This antibody-dependent enhancement is exploited in this experiment. Yamanaka and Konishi showed that the D4-I-1D6 monoclonal antibody enhance DENV-2 propagation in K562 cells [25]. In the presence of the antibody, nearly 100% of K562 cells were infected with DENV-2, yielding a concentration of 1 × 10^7.2^ FFU/ml, higher than the concentration reached without any antibody. When injected into an adult immuno-competent mouse, the antibody also enhanced the yield of DENV-2 in the periphery [26]. This is in contrast to mice that were injected with DENV-infected cells cultured without exogenous antibody; only very low level of circulating virus were detected [13].

In accordance with Yamanaka and Konishi [26], our infected C3H mice exhibited viremia 4–7 h post-injection. This viremia was higher than what was achieved with injection of DENV into IRF 3/7 knock-out mice [15]. Our model yielded a mean of 1.04 × 10^5^ PFU/ml (min. 4 × 10^4^ PFU/ml, max. 2.64 × 10^5^ PFU/ml), while IRF 3/7 knock-out mice only yielded a mean of 7.14 × 10^2^ PFU/ml (min. 3.0 × 10^1^ PFU/mL, max. 2.15 × 10^3^ PFU/mL). The same injection technique also worked with different adult immuno-competent mouse type, such as ICR [26].

Contrary to injected virus into immuno-compromised mice, the viremia reached in this technique is not due to replication of DENV inside the mouse. The infected cells leak the virus out into the mice’s bloodstream for at least until 7 hours post-injection. By maintaining the viremia at sufficient levels long enough for mosquitoes to acquire the virus, we managed to infect a large number of *Ae. aegypti* simultaneously by letting the mosquitoes to feed blood directly for 1 h.

In the present work, the existence of DENV-2 in mosquitoes was confirmed both by plaque assay and RT-PCR. Approximately 93% of tested mosquito samples in various time frames were positive for DENV-2. Furthermore, the concentration of DENV-2 in mosquitoes remained high at least until 12 h post-acquisition, with a mean of 8.92 × 10^3^ PFU/ml (min. 0, max. 2.64 × 10^4^ PFU/ml).

## Conclusions

We have developed a new technique to simultaneously infect a large number of *Ae. aegypti* by means of viremic immuno-compentent mouse. This new method simulates the natural route of mosquito infection with dengue virus. In this paper, we did not pursue the relationship between virus concentration and post-injection time with the virus concentration in mice or individual mosquitoes, issues that will be the subjects of our future experiments.

## Competing interests

The authors declare that there are no competing interests.

## Authors’ contributions

LR and YE carried out the infection. LR and YM confirmed the infection. EK, AY, YS, and YE conceived the study, participated in its design and coordination, and helped to draft the manuscript. TT, IK, and TK provided critical revisions. All authors read and approved the final manuscript.
